# Severe Influenza B-associated Encephalopathy With Vocal Cord Paralysis in an Otherwise Healthy Adolescent: A Case Report

**DOI:** 10.7759/cureus.86916

**Published:** 2025-06-28

**Authors:** Mahmoud Helaly, Mahmoud Ali, Mohamad Aly

**Affiliations:** 1 Critical Care Medicine, Manchester University NHS Foundation Trust, Manchester, GBR

**Keywords:** bilateral vocal cord, bilateral vocal cord paralysis, influenza b, neurocritical care, viral encephalopathy

## Abstract

Influenza B is generally considered a mild respiratory virus, but this case demonstrates its potential for severe neurological complications in otherwise healthy individuals. We describe a 17-year-old male patient who developed encephalopathy and bilateral vocal cord paralysis, requiring intensive care and tracheostomy. The patient gradually recovered following supportive care and multidisciplinary rehabilitation. This report highlights the importance of recognizing rare complications during influenza outbreaks.

## Introduction

Influenza viruses remain one of the most common causes of acute respiratory tract infections globally [1,2]. Seasonal epidemics are driven primarily by influenza A and B viruses, with influenza A responsible for most infections due to its higher mutation rate and pandemic potential. However, Influenza B, often considered milder, can also lead to significant complications, particularly in children, the elderly, and those with comorbidities [3].

Influenza-associated encephalopathy refers to brain dysfunction triggered by either direct viral invasion or the host immune response, presenting with seizures, altered mental status, or coma [4]. Vocal cord paralysis results from impaired function of the recurrent laryngeal nerves, potentially due to neuropathy, trauma, or viral inflammation.

Although influenza A has historically been associated with severe neurological complications, emerging evidence suggests that influenza B can also cause central nervous system (CNS) involvement, even in previously healthy individuals [5]. Studies estimate that neurological complications occur in approximately one in 1000 pediatric influenza cases, with influenza B contributing to a growing number of reports.

This case describes an adolescent who developed encephalopathy and bilateral vocal cord paralysis. This case underscores the importance of early identification, appropriate treatment, and coordinated multidisciplinary care in managing rare but serious influenza-related complications.

## Case presentation

A 17-year-old male patient with no prior medical conditions presented to the emergency department (ED) following a generalized tonic-clonic seizure witnessed at home. The episode lasted approximately 5-10 minutes, accompanied by tongue biting and urinary incontinence. Post-ictally, the patient was drowsy, irritable, and intermittently unresponsive. Concerned family members reported initiating chest compressions due to perceived unresponsiveness, although there was no confirmed cardiac arrest.

One day earlier, the patient had been seen in the ED for fever, myalgia, and poor oral intake persisting for a week. He was diagnosed with a viral illness and discharged with supportive care. A throat swab collected during that visit later tested positive for influenza B by polymerase chain reaction (PCR). There was no history of travel, exposure to sick contacts, or substance use.

On arrival, the patient was febrile (38.5°C), tachycardic (HR 126 bpm), and hypertensive (BP 142/113 mmHg), with an oxygen saturation of 99% on room air. Neurological assessment revealed a Glasgow Coma Scale (GCS) score of 9 (E3V1M5) and sluggish, reactive pupils (6-7 mm bilaterally), without any lateralizing signs. 

Multiplex PCR yielded negative results for herpes simplex virus (HSV), varicella-zoster virus (VZV), enterovirus, parechovirus, and influenza A/B.** **Despite a negative PCR panel for influenza A/B, HSV, VZV, enterovirus, and parechovirus in cerebrospinal fluid (CSF), a throat swab confirmed influenza B via RT-PCR on the initial ED visit. The negative CSF PCR may be attributed to timing, low viral load, or compartmentalization. Literature supports that CSF viral PCR can yield false negatives in up to 30% of influenza-associated encephalitis cases. Empirical IV acyclovir and oseltamivir were started due to clinical suspicion and supportive CSF findings (lymphocytic pleocytosis, elevated protein).

Levetiracetam was initiated for seizure prophylaxis. He was transferred to the intensive care unit (ICU) for airway protection and close monitoring due to fluctuating consciousness and agitation. Table 1 summarizes the patient's laboratory findings and corresponding reference ranges.

**Table 1:** Summary of laboratory findings and reference ranges Laboratory parameters collected during initial and subsequent assessments. WBC: white blood cell; ALT: alanine aminotransferase; CSF: cerebrospinal fluid

Parameter	Day 1 value	Reference range	Interpretation
WBC count	7.8×10⁹/L	4.0-11.0×10⁹/L	Normal
C-reactive protein	8 mg/L	<5 mg/L	Increased
Lactate	5.4 mmol/L	0.5-2.2 mmol/L	Increased
ALT	10 IU/L	7-56 IU/L	Normal
Albumin	41 g/L	35-50 g/L	Normal
CSF WBC	58 cells/µL	0-5 cells/µL	Increased
CSF protein	0.68 g/L	0.15-0.45 g/L	Increased
CSF glucose	4.1 mmol/L	2.2-3.9 mmol/L	Slightly increased

**Figure 1:**
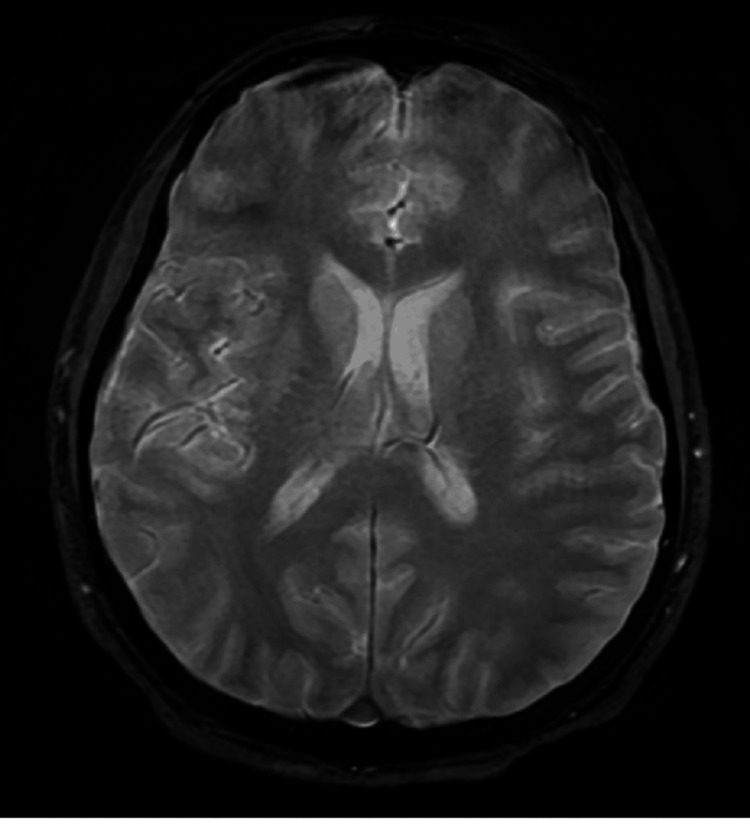
Axial T2-weighted brain MRI This axial T2-weighted image demonstrates structurally normal brain anatomy with no evidence of acute infarction, hemorrhage, or encephalitic changes. The ventricles appear normal in size and shape, with no signs of hydrocephalus or midline shift. Despite the absence of overt abnormalities, diagnostic interpretation was hindered by motion artifacts, likely due to the patient's reduced level of consciousness and need for sedation, limiting the sensitivity of the scan in detecting subtle parenchymal changes. In the context of the patient's clinical presentation, the normal MRI underscores the challenge of radiological diagnosis in viral encephalopathy and highlights the importance of EEG and CSF analysis in reaching a clinical diagnosis. MRI: magnetic resonance imaging; EEG: electroencephalogram; CSF: cerebrospinal fluid

A non-contrast brain computed tomography (CT) scan was unremarkable. Magnetic resonance imaging (MRI) showed no acute changes, although image quality was affected by motion artifacts, a common limitation in sedated or critically ill patients (Figure 1). An electroencephalogram (EEG) demonstrated diffuse slowing suggestive of moderate-to-severe encephalopathy without epileptiform discharges.

ICU course

**Figure 2:**
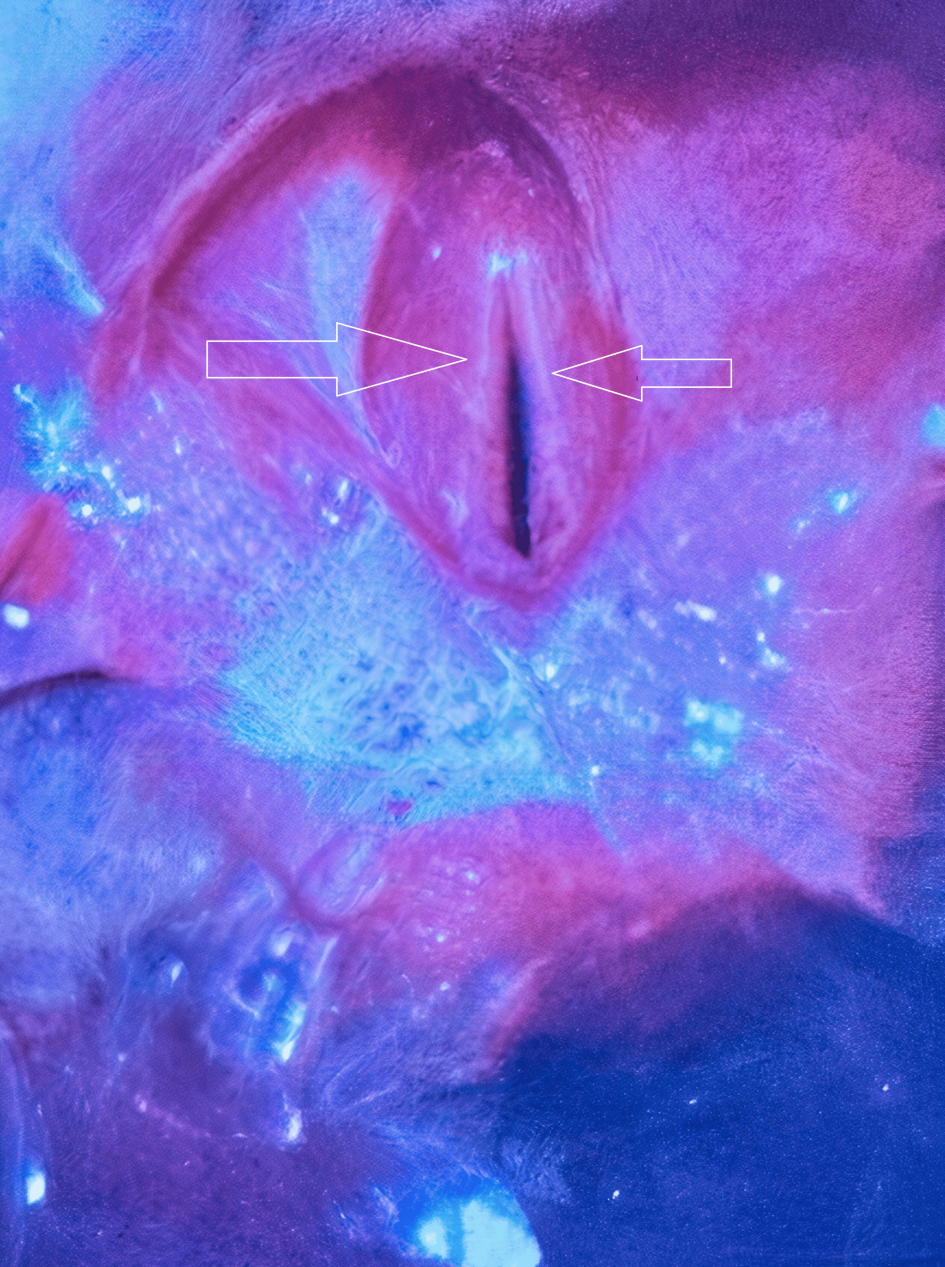
Endoscopic view of the larynx Flexible nasendoscopy showing bilateral vocal cords in a paramedian position, consistent with bilateral vocal cord paralysis. The glottic gap is minimal, contributing to airway obstruction and stridor during attempted extubation. No active mucosal lesions, edema, or granulation tissue are noted. This finding necessitated tracheostomy for airway protection and management.

The patient was intubated and sedated. Over the next two weeks, repeated attempts at extubation failed due to stridor, copious secretions, and airway compromise. Flexible nasendoscopy revealed bilateral vocal cord immobility in the paramedian position, consistent with bilateral vocal cord paralysis (Figure 2).

Empirical treatment included the following: oseltamivir 75 mg orally twice daily for five days, initiated promptly despite the initial CSF PCR negativity; IV acyclovir (10 mg/kg every eight hours), discontinued after HSV was ruled out; and levetiracetam 500 mg twice daily, continued for seizure prophylaxis and tapered on outpatient follow-up. Corticosteroids were not administered, as there was no radiological or clinical suspicion of autoimmune or demyelinating processes.

On ICU day 13, a percutaneous tracheostomy was performed to secure the airway and facilitate weaning. Additional investigations ruled out alternative causes of vocal cord dysfunction. These included the following: viral etiologies, with severe acute respiratory syndrome coronavirus 2 (SARS-CoV-2), Epstein-Barr virus (EBV), cytomegalovirus (CMV), and adenovirus negative on nasopharyngeal PCR; autoimmune screening, with NMDA-R, LGI1, CASPR2, GABA-B, and AMPA1/2 antibodies all negative; myasthenia gravis testing, with acetylcholine receptor antibodies negative; Guillain-Barré syndrome (GBS) mimics, with anti-ganglioside antibodies (GM1, GD1a, GQ1b) negative; and intubation-related trauma, with the patient intubated with a 7.5 mm endotracheal tube for 13 days and ENT evaluation and imaging ruling out pressure-induced injury as the primary cause.

**Figure 3:**
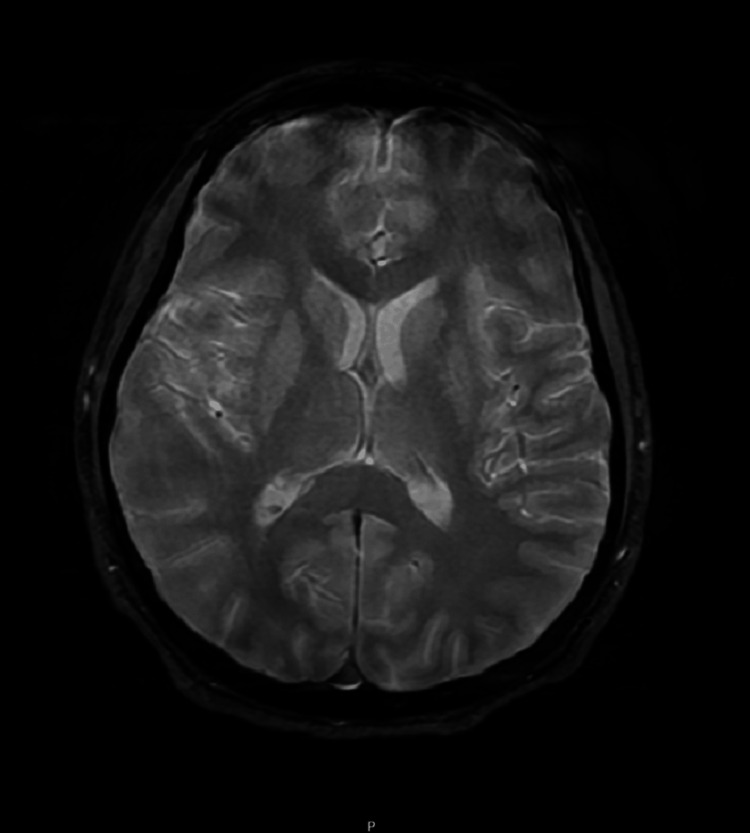
Axial T2-weighted brain MRI T2-weighted axial MRI demonstrating no acute abnormalities. Brain parenchyma appears structurally intact with normal ventricular size and no signs of edema, hemorrhage, or mass effect. Motion artifacts are noted, limiting sensitivity for subtle changes. MRI: magnetic resonance imaging

Repeat MRI again showed no acute pathology, although motion artifacts persisted (Figure 3). A follow-up EEG showed improving encephalopathy, with no evidence of seizure activity. Gradually, the patient's mental status improved. By day 30, he was awake, interactive, and using an electronic device to communicate.

Speech and Language Therapy (SLT) evaluation confirmed severe dysphagia with aspiration. Videofluoroscopy revealed persistent vocal cord dysfunction with incomplete glottic closure. Enteral nutrition continued via nasogastric tube. Over time, the patient transitioned to cuff-down tracheostomy during the day. ENT and SLT follow-ups showed improvement in swallow function despite ongoing vocal cord impairment. Antifungal therapy was initiated for oral candidiasis (Video 1).

**Video 1:** Videofluoroscopic swallow study demonstrating subglottic stenosis This videofluoroscopic swallowing study demonstrates significant subglottic stenosis contributing to airway compromise and aspiration risk. The narrowing is most consistent with post-intubation changes exacerbated by underlying neuromuscular dysfunction. The study informed the decision to maintain enteral nutrition and continue cuff-down tracheostomy management.

By day 47, the tracheostomy was decannulated successfully. Oral intake was reintroduced, starting with soft solids and thickened fluids. He remained seizure-free on levetiracetam.

The patient was discharged home on day 56 with outpatient follow-up arranged with the ENT, Neurology, and SLT teams. He was advised to avoid driving for six months and to taper antiepileptic medication over 3-6 months under neurological supervision.

## Discussion

Although influenza A is more commonly associated with neurological complications, influenza B has increasingly been implicated in CNS involvement, particularly in children and adolescents [1]. The pathogenesis of influenza-associated encephalitis may involve direct viral invasion or immune-mediated mechanisms [2].

In this case, CSF analysis showed lymphocytic pleocytosis and elevated protein levels, suggestive of viral etiology, despite negative PCR testing. Such false negatives may result from timing, low viral load, or sampling issues [3]. Thus, clinical judgment remains essential, and empiric antiviral therapy should not be withheld in suspected cases.

Bilateral vocal cord paralysis in the setting of viral encephalopathy is exceptionally rare. While influenza-associated cranial neuropathies have been documented, most involve facial nerve palsy or ophthalmoplegia. We found no published reports of bilateral vocal cord paralysis following influenza B in previously healthy adolescents.

A 2022 retrospective study reported neurological complications in 3.7% of pediatric influenza B cases, including seizures and altered mental status, but none involved vocal cord dysfunction. The absence of structural CNS damage and the gradual spontaneous recovery support a neuropathic, likely inflammatory etiology affecting the recurrent laryngeal nerves.

Possible mechanisms include viral neuropathy affecting the vagus or recurrent laryngeal nerves, critical illness polyneuropathy, and traumatic injury from intubation [4,5]. Given the patient's recovery without surgical intervention, a transient neuropathic process is likely.

MRI findings were inconclusive due to poor image quality, a recognized challenge in sedated or unstable patients [6,7]. EEG findings were consistent with viral encephalopathy and helped guide seizure management [8].

Successful recovery in this case was made possible by prompt ICU care, early antiviral treatment, and coordinated multidisciplinary support involving Neurology, ENT, SLT, and Nutrition teams.

Recent studies have underscored the neuroinvasive potential of influenza B, though most reports involve pediatric patients with underlying risk factors. The occurrence of bilateral vocal cord paralysis further expands the known spectrum of complications, aligning with reports that implicate neuropathic mechanisms, including inflammation of cranial nerves or direct viral neurotropism. This case supports a broader clinical suspicion for CNS involvement even in healthy individuals and suggests the need for further research into long-term neurological outcomes post-influenza infection.

## Conclusions

This case highlights the need for heightened vigilance during influenza seasons, even in otherwise healthy adolescents. The long-term implications of post-influenza neurological dysfunction, including cranial nerve involvement, warrant further study. Vaccination strategies may help mitigate the risk of rare but debilitating complications such as encephalopathy and vocal cord paralysis. Future cases should aim to capture detailed follow-up data on recovery, recurrence, and quality of life outcomes.

Clinicians should remain vigilant when assessing patients with altered mental status or respiratory symptoms during influenza outbreaks. Timely recognition, empirical treatment, and multidisciplinary care are critical to ensuring favorable outcomes in these complex cases.
